# New Sample Preparation Method for Quantification of Phenolic Compounds of Tea (*Camellia sinensis* L. Kuntze): A Polyphenol Rich Plant

**DOI:** 10.1155/2015/964341

**Published:** 2015-10-12

**Authors:** P. A. Nimal Punyasiri, Brasathe Jeganathan, Jeevan Dananjaya Kottawa-Arachchi, Mahasen A. B. Ranatunga, I. Sarath B. Abeysinghe, M. T. Kumudini Gunasekare, B. M. Rathnayake Bandara

**Affiliations:** ^1^Institute of Biochemistry Molecular Biology and Biotechnology, University of Colombo, 00300 Colombo, Sri Lanka; ^2^Department of Food Science and Technology, Faculty of Agriculture, University of Peradeniya, 20400 Peradeniya, Sri Lanka; ^3^Tea Research Institute of Sri Lanka, 22100 Talawakelle, Sri Lanka; ^4^Coordinating Secretariat for Science, Technology & Innovation, 3rd Floor, Standard Charted Building, Janadhipathi Mawatha, 00100 Colombo, Sri Lanka; ^5^Department of Chemistry, Faculty of Science, University of Peradeniya, 20400 Peradeniya, Sri Lanka

## Abstract

Chemical analysis of the Sri Lankan tea (*Camellia sinensis*, L.) germplasm would immensely contribute to the success of the tea breeding programme. However, the polyphenols, particularly catechins (flavan-3-ols), are readily prone to oxidation in the conventional method of sample preparation. Therefore, optimization of the present sample preparation methodology for the profiling of metabolites is much important. Two sample preparation methodologies were compared, fresh leaves (as in the conventional procedures) and freeze-dried leaves (a new procedure), for quantification of major metabolites by employing two cultivars, one is known to be high quality black tea and the other low quality black tea. The amounts of major metabolites such as catechins, caffeine, gallic acid, and theobromine, recorded in the new sampling procedure via freeze-dried leaves, were significantly higher than those recorded in the conventional sample preparation procedure. Additionally new method required less amount of leaf sample for analysis of major metabolites and facilitates storage of samples until analysis. The freeze-dried method would be useful for high throughput analysis of large number of samples in shorter period without chemical deterioration starting from the point of harvest until usage. Hence, this method is more suitable for metabolite profiling of tea as well as other phenol rich plants.

## 1. Introduction

Phenolic compounds are the most commonly studied of all secondary metabolites because of their significant concentration and their significant roles in plant tissues [1, 2]. This highly diverse group of secondary metabolites widely is distributed in vegetable foods (legumes, cereals, and fruits) and beverages (tea, cider, and wine), which are important constituents of the human diet [3].

The rapid degradation of flavonoids during the extraction is a major drawback for the accurate quantification of this ubiquitous group of compounds. Because of their importance as potent antioxidants molecules, quantification flavonoids in fruits, vegetable, and other crops are important [4]. They become perishable mainly due to the enzymatic oxidation regulated by the enzyme polyphenol oxidase and light [5, 6]. Polyphenol oxidase is activated when the plant tissues are macerated and the enzymes get mixed with the substrate and the major parts of the native flavonoids are destroyed before quantification.

The sustainability of the tea industry mainly relies on developing new tea (*Camellia sinensis*, L.) cultivars incorporated with genetic resistance to biotic and abiotic stresses and capable of producing diverse tea products of desired character. The quality of tea product depends on the chemical composition of fresh tea leaves [7] and the method of manufacture [8]. The chemical composition of tea leaves is determined by the inherent genetic attributes of the accession [9] and geoclimatic factors [10].

Currently about 600 germplasm accessions are being maintained in the field gene bank of the Tea Research Institute of Sri Lanka (TRI) and these accessions are not adequately evaluated for biochemical, agronomic, and molecular traits [11, 12]. Most of the earlier attempts concentrated on single metabolite variation of tea cultivars, and work on measurement of a broad range of metabolites is relatively limited [13].

Thus, metabolite profiling of representative accessions from the Sri Lankan tea germplasm by chemical analysis of tea leaves would immensely contribute to the success of a tea breeding programme. The polyphenols, particularly catechins (flavan-3-ols) and their oxidation products, which are eventually responsible for the quality of tea [14], are readily oxidized by polyphenol oxidase enzymes. The collection, handling, and storage of a large number of leaf samples for metabolite profiling thus require a sample preparation procedure that would minimize the oxidation of polyphenols and formation of artifacts. In this backdrop, this research has been carried out with the objective to optimize the sample preparation procedure to minimize the chemical deterioration that takes place in tea flush due to the inevitable delay in analysis. For this purpose, a comparative study has been conducted between the fresh leaves (conventional procedures) and freeze-dried leaves (new sampling method), for quantification of some critical metabolites—gallic acid, catechins, and methylxanthines, by employing two cultivars, one (DT1) of which is known to yield high quality black tea and the other (TRI2025) poor quality black tea.

## 2. Materials and Methods

### 2.1. Standards and Solvents

Caffeine, gallic acid, (−)-epicatechin, (−)-epigallocatechin, (−)-epigallocatechin gallate, and (−)-epicatechin gallate were purchased from Sigma chemical Co. (St. Louis, MO, USA). Methanol, acetonitrile, acetic acid, and Folin-Ciocalteu's phenol reagent were purchased from BDH, UK. Standard reference material (SRM 3254) for green tea was obtained from the National Institute of Standards and Testing (NIST), USA. All reagents were HPLC grade or higher. A Millipore Milli-Q water purification system was used to obtain high purity of water.

### 2.2. Plant Materials and Experimental Location

Two cultivars (DT1 and TRI2025) were selected for this study from* ex situ* field gene bank at the Tea Research Institute of Sri Lanka, Talawakelle (latitude 6°54′N, longitude 80°42′E). The plants were of same age and have been maintained under similar growth conditions recommended by the Tea Research Institute of Sri Lanka.

### 2.3. Extraction of Fresh Tea Leaves (Conventional Procedure)

Five grams of fresh two leaves and a bud was placed in 70% aqueous methanol (40 mL) at boiling temperature for 10 min. The mixture was cooled to room temperature and homogenized for 3 min using a top-drive macerator (Ultra-Turrax, USA). The homogenate was centrifuged (24.15 ×g for 15 min) and the supernatant was decanted into a 50 mL volumetric flask. The residue was reextracted following the above extraction procedure with 70% aqueous methanol (10 mL) at boiling temperature. The volume of the pooled extract was made up to 50 mL with 70% aqueous methanol. An aliquot (1.0 mL) of the above methanol extract was transferred into a 200 mL volumetric flask and topped up with Milli-Q water.

### 2.4. Preparation of Freeze-Dried Tea Sample (New Procedure)

Approximately 10 g of fresh two leaves and an active bud from each cultivar was harvested and brought to the laboratory in ice box at 4°C. The plant material was immediately deep frozen at −80°C for 6 h and then freeze-dried for 24 h (Labconco-Freezone, USA). The freeze-dried flush was ground to a fine powder (passed through 500 *μ*m mesh) in a laboratory mill, sealed in triple laminated aluminum foil packages, and stored until use at room temperature.

### 2.5. Extraction of Freeze-Dried Tea Flush

0.2000 ± 0.001 g freeze-dried tea sample was weighed into a centrifugation tube and extracted with 5.0 mL of 70% methanol/water extraction mixture, preheated to 70°C for half an hour. Heating of the test portions in the water bath was continued for 10 min with mixing on the vortex mixer at the beginning, after 5 min and at the end of 10 min. Then the extraction tube was allowed to cool to room temperature and placed in a centrifuge at 1095 ×g for 10 min. The supernatant was carefully decanted into 10 mL volumetric flask. The extraction steps were repeated on the residue, the extracts were combined, and the volume was made up to 10 mL with cold methanol/water extraction mixture and mixed. 1.0 mL of the sample extract was topped up to 5 mL with freshly prepared stabilizing solution containing 500 *μ*g mL^−1^ of EDTA and 500 *μ*g mL^−1^ ascorbic acid in 10% volume fraction of acetonitrile. An aliquot was filtered through 0.45 *μ*m regenerated cellulose sample filter. 10.0 *μ*L of the sample was used for the HPLC analysis. All samples were analysed in triplicate.

### 2.6. Determination of Dry Matter Content

The dry matter content was determined by loss in mass at 103°C determined on a portion of the test sample in accordance with ISO1573 [15] method for both fresh flush leaves and freeze-dried flush.

### 2.7. Determination of Total Polyphenols (TPP) in Fresh Flush and Freeze-Dried Flush Extracts

A series of gallic acid standard solutions was prepared with concentrations ranging from 10 to 50 *μ*g mL^−1^. The TPP in the standards and the extracts were determined in quadruplicate using Folin-Ciocalteu's phenol reagent according to ISO 14502-1 [16] method using a spectrophotometer (CARY 50Bio, Varian, USA) set at 765 nm.

### 2.8. Analysis of Metabolites in Fresh Leaf and Freeze-Dried Extracts Using High Performance Liquid Chromatography (HPLC)

Caffeine, EC, ECg, EGC, EGCg, gallic acid, and theobromine were quantified in duplicate in two trials of samples using ISO 14502-2 [17] method by HPLC (Agilent 1260 HPLC Infinity System) on a phenyl-bonded column using elution with UV detection at 278 nm.

### 2.9. HPLC Analysis

#### 2.9.1. Instrumentation

The HPLC instrument consisted of Agilent 1260 Infinity HPLC system with solvent delivery system, temperature-controlled column oven, autosampler, degasser unit, and a photodiode array detector and Open Lab/Chemstation software system.

#### 2.9.2. Adjustment of the Apparatus

Luna-5 *μ*m Phenyl-Hexyl-4.6 × 250 mm column (Phenomenex Inc., USA) with a guard column made of the same material (Security-Guard, Phenomenex Inc., USA) was used. Mobile phase A was 9% acetonitrile containing 2% acetic acid (BDH Hipersolv-HPLC grade). Mobile phase B was 80% aqueous acetonitrile (BDH Hipersolv-HPLC grade). Solvent gradient was reset to 100% mobile phase A and allowed to equilibrate for 10 mins before the next injection. The elution was reset to 100% mobile phase A and allowed to equilibrate for 10 min before next injection.

Flow rate was set to 1.00 mL/min and column oven temperature was kept at 35 ± 0.5°C. The injection volume was 10 *μ*L. The metabolites were detected at 278 nm. Once the flow rate of the mobile phase and temperature were stable, the column was conditioned with a blank gradient run. After each batch of analysis, the chromatographic system and column were thoroughly flushed with 50% aqueous acetonitrile and column sealing plugs were replaced if disconnected for storage.

### 2.10. Identification and Quantification of Gallic Acid, Catechins, and Methylxanthines

The individual catechins (EC, ECg, EGC, and EGCg) and gallic acid were identified by comparing retention times from sample chromatograms with those recorded for a variety of standards obtained under the same chromatographic conditions. Calibration curves were constructed for the individual compounds. The use of diode array detection allowed the UV profile of the catechin peaks to be scrutinized and peak purity to be assessed, which was particularly useful for the determination of the low levels of catechins in tea cultivars.

Alternatively, caffeine was used as a standard in conjunction with individual catechins considering the Relative Response Factors (RRFs) established by an ISO international interlaboratory test. Data was collected using the data collection/integration system for all peaks in the extracts.

### 2.11. Validation Procedure

Standard reference material (SRM 3254) for green tea certified by NIST, USA, was used for the validation of the analytical methodology.

### 2.12. Statistical Analysis

Completely Randomized Design (CRD) was used as the experimental model. Significant differences (*P* < 0.01) were tested using two tailed paired *t*-test at 95% confidence level. Least Significant Difference (LSD) was used as the mean separation technique. All these analyses were carried out with the statistical analysis software package (SAS Version. 9.1 for Windows, SAS Institute Inc., USA).

## 3. Results and Discussion

A major problem in the quantification of catechins is their highly perishable nature. One of the most important fractions in tea flush is the proteins, because they include the enzymes which are required for the metabolism in the tea plant and for black tea manufacture [7]. Polyphenol oxidase is an important enzyme available in tea, which is activated by light [18] and is responsible for the oxidation of flavonols in the presence of atmospheric oxygen. In the conventional sample preparation methodology there is an inevitable delay in the analysis during which the tea flush is subjected to mechanical damage. This results in the formation of characteristic compounds of black tea [19, 20]. Though it is a positive reaction initiated in the rolling process of tea manufacturing, it hinders accurate and precise measurement of metabolites in the tea flush. Further, the content of catechins is dependent upon other factors including particularly light since some of the important enzymes of the flavonoid biosynthetic pathway are activated by light [21]. Since the analysis of the batch of cultivars sampled has to be done on the same day, it was not possible to handle a large number of samples at a time.

**Figure 1:**
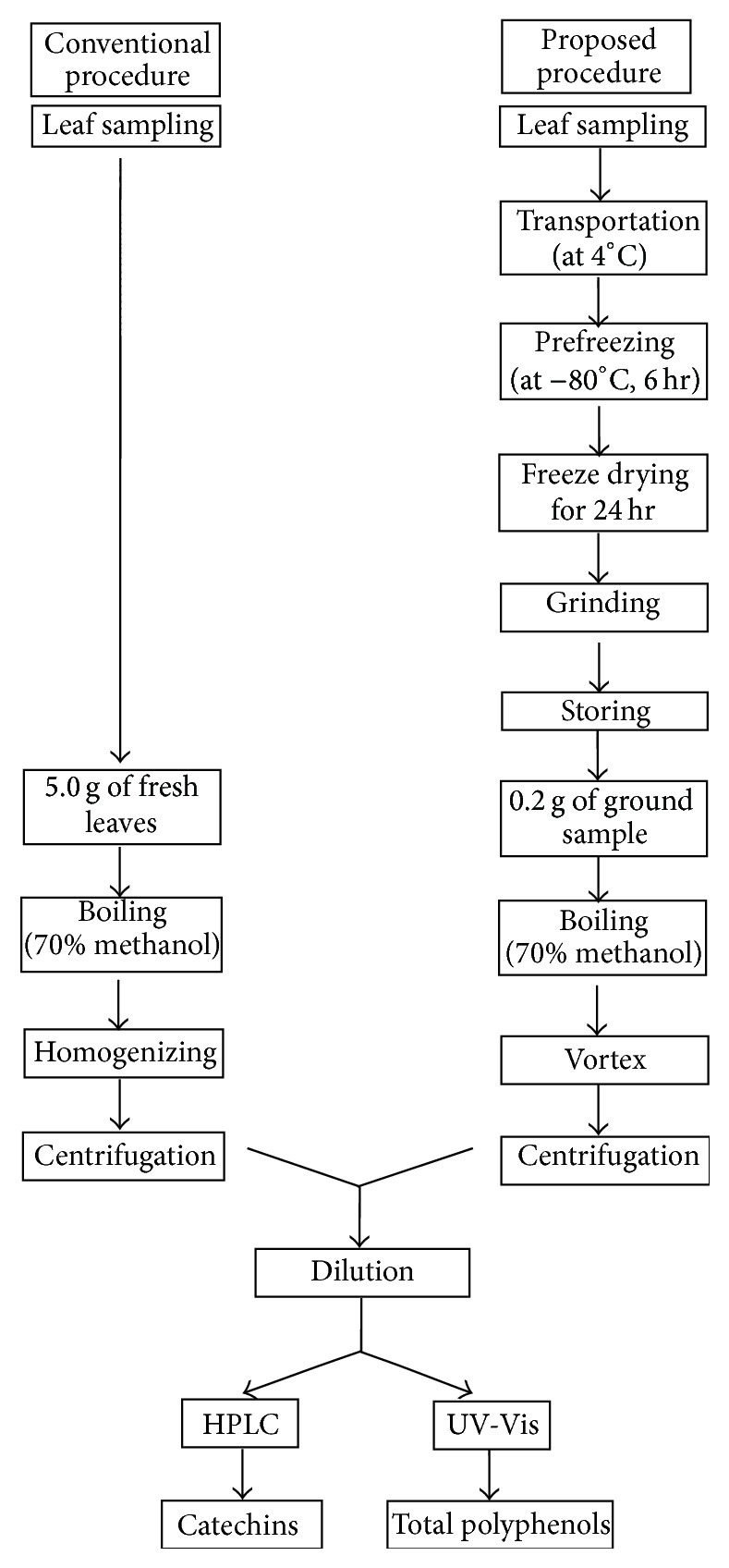
Steps of conventional and new sample preparation procedure for metabolite analysis of tea.

In contrast, the new method suppressed chemical deterioration starting from the point of harvest until usage. As described in Figure 1, the samples harvested from the field were immediately collected into and ice box at 4°C whereby the chemical deterioration of the metabolites was minimized. Prefreezing at −80°C for 6 h to two weeks is a convenient first step in sample preparation prior to freeze-drying. Freeze-drying is a preservation technique by which moisture is removed and the shelf-life is increased approximately up to one year. The dry matter content of the freeze-dried samples was eight times less than that of the fresh flush. Therefore, substantially less amount of sample was required for analysis in the new method (Table 1) and convenient handling of many samples was possible at a time. Also, freeze-dried leaf samples can be stored for a long time when packed in triple laminated aluminum sheets and it facilitates sampling at once and stored for metabolite analysis. Thus, environmental influence on metabolite availability could be minimized specially when handling large number of samples.

**Figure 2:**
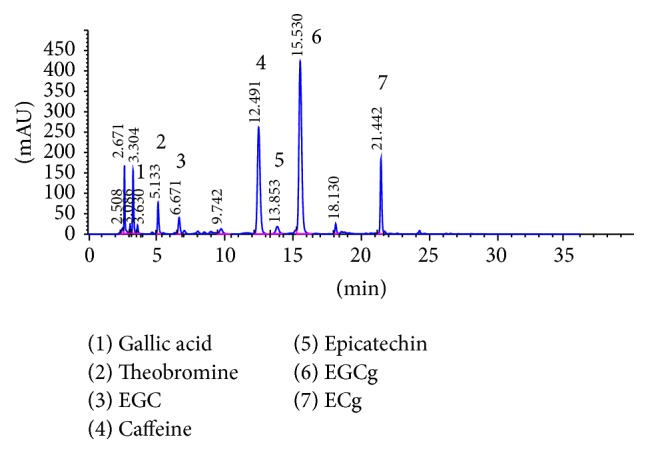
HPLC chromatogram of representative tea accession quantified using ISO: 14502-2 method.

Maximum recovery of the metabolites required a minimum of 70% methanol in the extractant which was sufficient to inactivate polyphenol oxidase [22]. Multiple extraction procedures of short durations (10 min) at moderate temperature (70°C) in the new method were more favourable than those involving boiling temperature and long extraction times in a macerator as such conditions would lead to decomposition of catechins [23]. Efficient separation of caffeine, (−)-epicatechin (EC), (−)-epigallocatechin (EGC), (−)-epigallocatechin gallate (EGCg), (−)-epicatechin gallate (ECg), gallic acid, and theobromine in the tea extracts (Figure 2) was achieved in less than 25 min of a chromatographic run by the gradient elution with acetonitrile, water, and acetic acid. All calibration curves displayed excellent correlation coefficients (≥0.998). Although the sensitivity of the analysis can be increased by threefold by using a shorter detection wavelength, the chance of interference by other components present in the tea extract is high [23]. Hence, 278 nm was used as the detector wavelength.

The Folin-Ciocalteu's phenol reagent used for TPP analysis contains phosphotungstic acid as oxidants, which on reduction yield a blue colour with a broad maximum absorption at 765 nm. As this reagent reacts with a wide range of polyphenol compounds, it is often used to estimate the “total polyphenol” content in plant material rich in polyphenols, although the response can vary with the individual components depending on their chemical structures. However, the Folin-Ciocalteu's reagent could simultaneously oxidize several nonphenolic organic compounds as well as some inorganic substances to give elevated apparent phenolic content [24]. As it is based on the reduction properties of sample components, it really measures the total reducing capacity of samples.

The mean values of TPP, and the amounts of four catechins (EC, ECg, EGC, and EGCg), caffeine, theobromine, and gallic acid recorded for the two cultivars, DT1 and TRI2025, corresponding to the two sampling procedures (fresh leaves and freeze-dried leaves), are given in Table 2. The concentration of each metabolite varied with the cultivar. For both cultivars, DT1 and TRI2025, the contents of all the metabolites (TPP, EC, ECg, EGC, EGCg, caffeine, theobromine, and gallic acid) determined by employing new method were found to be higher than in the conventional method; the difference was statistically significant (*P* < 0.05) for each of the metabolites except for theobromine in cultivar DT1.

National Institute of Standards and Testing (NIST) certified SRM 3254 quantified* via* these two methods confirms that the new method is accurate and precise for the determination of the major metabolites in tea.

## 4. Conclusion

The freeze-dried sampling procedure recorded significantly higher amount of metabolites with small leaf quantity and facilitates storage of samples without chemical deterioration starting from the point of harvest until usage. Hence, the method is more suitable for metabolite profiling of tea germplasm using high throughput analysis where large number of samples need to be handled.

## Figures and Tables

**Table 1:** Comparison of dry matter % and the sample required for analysis of cultivars DT1 and TRI2025.

Cultivars	Sample	Dry matter (%)	Sample required for analysis (g)
DT1	Fresh	20.38	5.00
	Freeze-dried	91.18	0.20

TRI2025	Fresh	21.41	5.00
	Freeze-dried	93.49	0.20

**Table 2:** Metabolite composition of tea flush of cultivars DT1 and TRI 2025 determined using two methods of sampling with fresh tea flush (conventional procedure) and freeze-dried tea flush (new procedure).

Metabolites	DT1 (mg g^−1^)					TRI 2025 (mg g^−1^)				
	Fresh	Freeze-dried	*P*	LSD	CV	Fresh	Freeze-dried	*P*	LSD	CV
TPP^††^	266.49 ± 2.79	282.27 ± 3.77	*∗∗*	3.56	1.21	266.33 ± 2.72	278.07 ± 3.54	*∗∗*	3.39	1.16
EC^†^	15.88 ± 0.29	19.27 ± 0.27	*∗∗*	0.48	1.59	6.27 ± 0.54	7.47 ± 0.41	*∗*	0.82	6.9
ECg^†^	37.65 ± 1.22	41.43 ± 0.53	*∗*	1.63	2.38	25.30 ± 1.45	30.71 ± 0.49	*∗∗*	1.87	3.86
EGC^†^	33.99 ± 0.91	42.21 ± 0.56	*∗∗*	1.31	1.99	17.54 ± 1.06	20.01 ± 0.92	*∗*	1.72	5.28
EGCg^†^	84.00 ± 5.15	93.61 ± 1.14	*∗*	6.45	4.20	104.17 ± 3.22	128.95 ± 2.33	*∗∗*	4.87	2.41
Caffeine^†^	35.31 ± 1.36	38.67 ± 0.33	*∗*	1.71	2.67	34.52 ± 0.13	41.71 ± 0.59	*∗∗*	0.74	1.12
Theobromine^†^	2.17 ± 0.15	2.25 ± 0.02	NS	0.18	4.90	4.62 ± 0.32	5.89 ± 0.17	*∗∗*	0.45	4.91
Gallic acid^†^	0.29 ± 0.01	0.55 ± 0.03	*∗∗*	0.03	4.26	0.31 ± 0.01	0.83 ± 0.01	*∗∗*	0.02	1.59

Means ± SD (*n* = 8), *P*: probability, ^*∗*^significant at 5% level of probability, ^*∗∗*^significant at 1% level of probability, NS: not significantly different, CV: coefficient of variance, LSD: Least Significant Difference, ^††^determined by ISO-14052-1 Spectrophotometric method, ^†^determined by ISO-14052-2 HPLC method, and TPP: total polyphenols.
